# Identification of Factors That Determine Adherence for Total Neoadjuvant Therapy in Locally Advanced Rectal Cancer

**DOI:** 10.3390/cancers18172853

**Published:** 2026-09-03

**Authors:** Cynthia Araradian, Emmett Hunnicutt, Siting Chen, Shawna Rivedal, Shelby Willis, Maura Walsh, Vassiliki Liana Tsikitis, Sandy Hwang Fang

**Affiliations:** 1 Department of Surgery, Oregon Health & Science University, Portland, OR 97239, USA; rivedal@ohsu.edu (S.R.); willshel@ohsu.edu (S.W.); walshma@ohsu.edu (M.W.); tsikitis@ohsu.edu (V.L.T.); fangs@ohsu.edu (S.H.F.); 2 School of Medicine, Oregon Health & Science University, Portland, OR 97239, USA; hunnicue@ohsu.edu; 3 Biostatistics and Design Program, Oregon Health & Science University, Portland, OR 97239, USA; chensi@ohsu.edu

**Keywords:** rectal cancer, locally advanced rectal cancer, LARC, TNT, chemotherapy, chemoradiation, neoadjuvant

## Abstract

**Background**: Locally advanced rectal cancer (LARC) treatment has shifted from the utilization of neoadjuvant chemoradiation (NACRT), followed by total mesorectal excision (TME) and adjuvant chemotherapy (AC) to the implementation of total neoadjuvant therapy (TNT). This transition was influenced by data from multiple studies reporting suboptimal compliance rates to adjuvant chemotherapy. As a result, a paradigm shift to TNT occurred, consisting of chemoradiation with chemotherapy, based on the RAPIDO and PRODIGE-23 studies. **Methods**: Following IRB approval, a single-institution retrospective chart review was conducted at a tertiary care academic institution for patients with LARC. Demographic, clinical, and treatment data were collected using REDCap. Patients were excluded if under 18 years of age, pregnant, or had stage 1 or metastatic rectal cancer. The primary objective was to evaluate TNT adherence; secondary objectives assessed adherence associations with demographic and clinical variables. **Results**: Two hundred twenty-nine patients with rectal cancer were initially included in the database, including 153 patients with LARC from 2019 to 2024. One hundred twenty-seven patients underwent TNT with an adherence rate of 87.5%. Statistically significant variables that contributed to adherence include male gender (*p* = 0.04) and non-use of recreational drugs (*p* = 0.04). **Conclusions**: This is the first real-world study assessing TNT adherence. The observed 87.5% adherence rate represents a significant improvement over historical data in which patients were treated with NACRT/TME/AC. Statistically significant variables that contributed to TNT adherence included male gender and non-use of recreational drugs. These factors may provide insight into gender differences and behavioral patterns that put patients in an at-risk category for non-adherence.

## Simple Summary

The management of locally advanced rectal cancer has evolved over the last several decades with a shift to total neoadjuvant therapy, in which patients complete chemoradiation and chemotherapy initially. After completing both therapies, patients either enter a watch-and-wait program or undergo surgery. This shift was adopted due to poor adherence rates to complete prior treatment regimens. The goal of our study was to determine adherence to the planned total neoadjuvant treatment course and identify patient characteristics that may influence patient adherence. With this research, we hope physicians can utilize this data to better counsel patients and use individual patients’ characteristics to identify the best treatment course for a patient with locally advanced rectal cancer.

## 1. Introduction

Locally advanced rectal cancer (LARC) management has evolved over the last several decades. The initial standard of care management for LARC consisted of neoadjuvant chemoradiation (NACRT), followed by surgery with total mesorectal excision (TME)-resection of the rectum and its surrounding mesorectum along embryological planes-followed by adjuvant chemotherapy (AC). The role of NACRT was to downsize and downstage the tumor and to help decrease local recurrence within the pelvis [1]. The addition of neoadjuvant chemotherapy to preoperative radiation helped as a radiosensitizing agent and overall did not contribute to significant changes in adherence to treatment [1]. Adjuvant therapy decreased the risk of developing distant metastases.

Historically, studies have demonstrated poor compliance rates of 40–57% for completing adjuvant chemotherapy. Reasons for this include postoperative complications and increased frailty that preclude moving forward to chemotherapy. In the Chronicle trial, 113 LARC patients received six courses of adjuvant oxaliplatin and capecitabine for 4 months versus observation alone following treatment with NACRT and surgical resection. The compliance rate was 48% in the AC group with no statistically significant benefit in disease-free survival (DFS) [2]. The EORTC 22921 study included 1011 patients assigned to preoperative radiation with or without neoadjuvant chemotherapy followed by surgery and then AC vs. observation. Of the patients assigned to AC, 43% were compliant, and there was no overall benefit for overall survival or disease-free survival [3]. In an Italian retrospective, multi-institutional study, 56% of patients completed adjuvant chemotherapy [4].

To improve the observed lower adherence rates for AC completion, Garcia-Aguilar et al. [5] completed a phase 2, non-randomized clinical trial assessing pathological complete response in patients who received neoadjuvant chemotherapy after NACRT. There was a statistically significant increase in complete pathological response after 6 cycles of fluorouracil (5-FU), leucovorin, oxaliplatin (FOLFOX) compared to 0, 2, or 4 cycles of FOLFOX [5]. This study laid the groundwork for the possibility of organ preservation. The importance and utility of total neoadjuvant therapy (TNT) were evaluated by the RAPIDO and PRODIGE-23 trials.

The RAPIDO multicenter, phase 3 randomized controlled trial compared standard-of-care long-course NACRT/TME/AC with the experimental group of short-course chemoradiation followed by six cycles of capecitabine and oxaliplatin (CAPOX) or nine cycles of FOLFOX followed by surgery. The experimental group demonstrated a pathologic complete response rate of 28.0% vs. 14% in the standard of care group, as well as significantly lower distant metastases with no statistical significance for the locoregional failure [6]. The UNICANCER-PRODIGE 23 trial utilized standard of care treatment of NACRT/TME/AC versus the experimental group of six cycles of neoadjuvant 5-FU, oxaliplatin, leucovorin, irinotecan (FOLFIRINOX), chemoradiation with capecitabine, TME, then AC. In the experimental group, 92% of patients completed pre-operative chemotherapy, with 22% of patients in the experimental group completing adjuvant chemotherapy in the first 3 months and 51% of the standard-of-care patients. Patients in the experimental group had a 3-year DFS of 76% vs. 69% and a significant improvement in the pathologic complete response rates [7]. Table 1 summarizes the aforementioned clinical trials.

**Figure 1:**
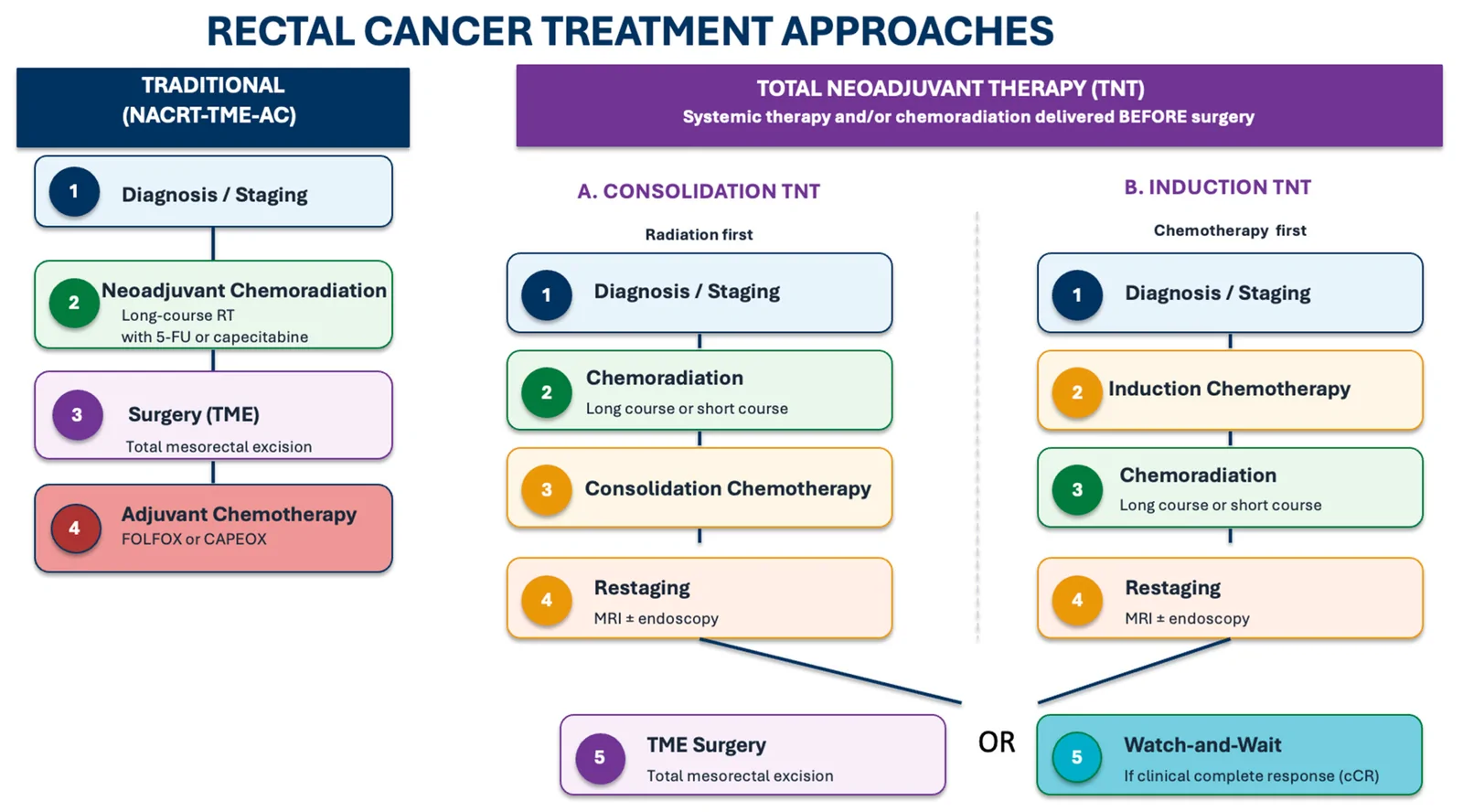
Rectal cancer treatment algorithms. The traditional approach included neoadjuvant chemoradiation (NACRT) followed by surgery with a total mesorectal excision (TME), then adjuvant chemotherapy (AC). Total neoadjuvant therapy (TNT) includes consolidation or induction chemotherapy, as seen in the figure above. The pathways converge with restaging to determine the next step of surgery or inclusion in watch and wait. Image created in PowerPoint.

Given the benefit seen with the use of neoadjuvant chemotherapy, TNT was implemented as the new standard of care for LARC. TNT includes chemoradiation as either short-course or long-course radiation and systemic chemotherapy given either before (induction) or after (consolidation) chemoradiation. This approach is followed by either a surgical intervention with TME, if there is an incomplete response, or organ preservation with a watch-and-wait approach for patients who have a complete response. The response is assessed with digital rectal examination, imaging, and endoscopic examination within 4–8 weeks of TNT [8]. The algorithm of the traditional approach to rectal cancer treatment compared to TNT is depicted in Figure 1. The consensus is that chemoradiation therapy serves to downgrade the size of the cancer, while induction chemotherapy is utilized when there are high-risk features for distant metastases, such as extramural vascular invasion.

Since one factor that influenced this paradigm shift was poor adherence to AC, we decided to evaluate rates of adherence with TNT, as there is a lack of real-world data evaluating this aspect of care. We conducted a retrospective analysis of our institution’s LARC patients who received TNT to assess rates of adherence to TNT completion and identify influential sociodemographic and treatment risk factors.

## 2. Materials and Methods

After IRB approval (#25714), patients diagnosed and treated for rectal cancer at a single tertiary care academic institution were retrospectively reviewed. Patients were included in this study if they were presented at the institution’s multidisciplinary tumor board conference; a list of patients diagnosed with rectal cancer and discussed in the multidisciplinary setting was obtained. Patients included were >18 years of age, had to have a diagnosis of rectal adenocarcinoma, and were found to have locally advanced rectal cancer (LARC) or stage 2 (T3N0) or 3 (node-positive) disease based on preoperative imaging. Retrospective chart review was performed to confirm rectal cancer diagnosis via endoscopic, imaging, and pathological evaluation. Patient demographic and cancer data were collected. This data included race, age, gender, co-morbid conditions, primary treatment facility, tumor stage, recurrence, cancer treatment including chemotherapy and radiation regimen, and surgical procedures. There was a special focus on understanding whether the treatment was given as a neoadjuvant and/or adjuvant regimen, whether treatment was completed, and if there was an effect due to a patient’s home location from their primary treatment hospital and whether the treating hospital was an academic or community site.

Exclusion criteria included age <18 years, pregnancy, stage 1 disease, and patients with distant metastasis.

Patients were either referred to this single academic tertiary care institution for a second opinion or received all or part of their primary treatment at this institution.

The study data were collected and managed utilizing Research Electronic Data Capture (REDCap), an electronic data capture tool hosted at Oregon Health and Science University [9].

The primary objective was to investigate the percentage of our institution’s locally advanced rectal cancer patients’ adherence to TNT. Adherence is defined as adherence to the specific treatment regimen instituted by the treating physician and completing the course of therapy, both chemoradiation and chemotherapy. Adherence to a treatment regimen included dose reduction and a regimen change due to patient intolerance or side effects. Patients were non-adherent if cycles were omitted from the originally planned treatment regimen or treatment was stopped. The secondary objective was to determine if the variables of patient characteristics (such as race, gender, age, etc.) and the location of their treating institution factored into adherence with TNT. For the patient characteristics addressed in this study, race was only included if reported by the patient in the electronic health record. Eastern Cooperative Oncology Group (ECOG) status was obtained from the medical oncology initial evaluation notes. Recreational drug use was obtained from notes when a patient reported using marijuana, heroin, methamphetamines, fentanyl, or misuse of prescribed drugs. The drug use criteria were defined as current recreational drug use, and a prior substance use history was not taken into consideration. Albumin and CEA were lab values obtained prior to the initiation of the treatment regimen.

### Statistical Analysis

IBM SPSS Statistics software (version 30.0 for Mac, IBM Corporation, Armonk, NY, USA) was employed to compute frequency distributions and descriptive statistics for each demographic variable.

Continuous variables were summarized as mean (SD) or median (IQR), and categorical variables as *n* (%). The differences in continuous variables were assessed using *t*-tests or Mann–Whitney U (rank-sum) tests when appropriate, and the differences in categorical variables were assessed using chi-square tests or Fisher’s exact tests when appropriate. A *p*-value < 0.05 was considered statistically significant. The statistical analyses were performed in STATA/SE (version 16.1, StataCorp LLC, College Station, TX, USA).

## 3. Results

**Figure 2:**
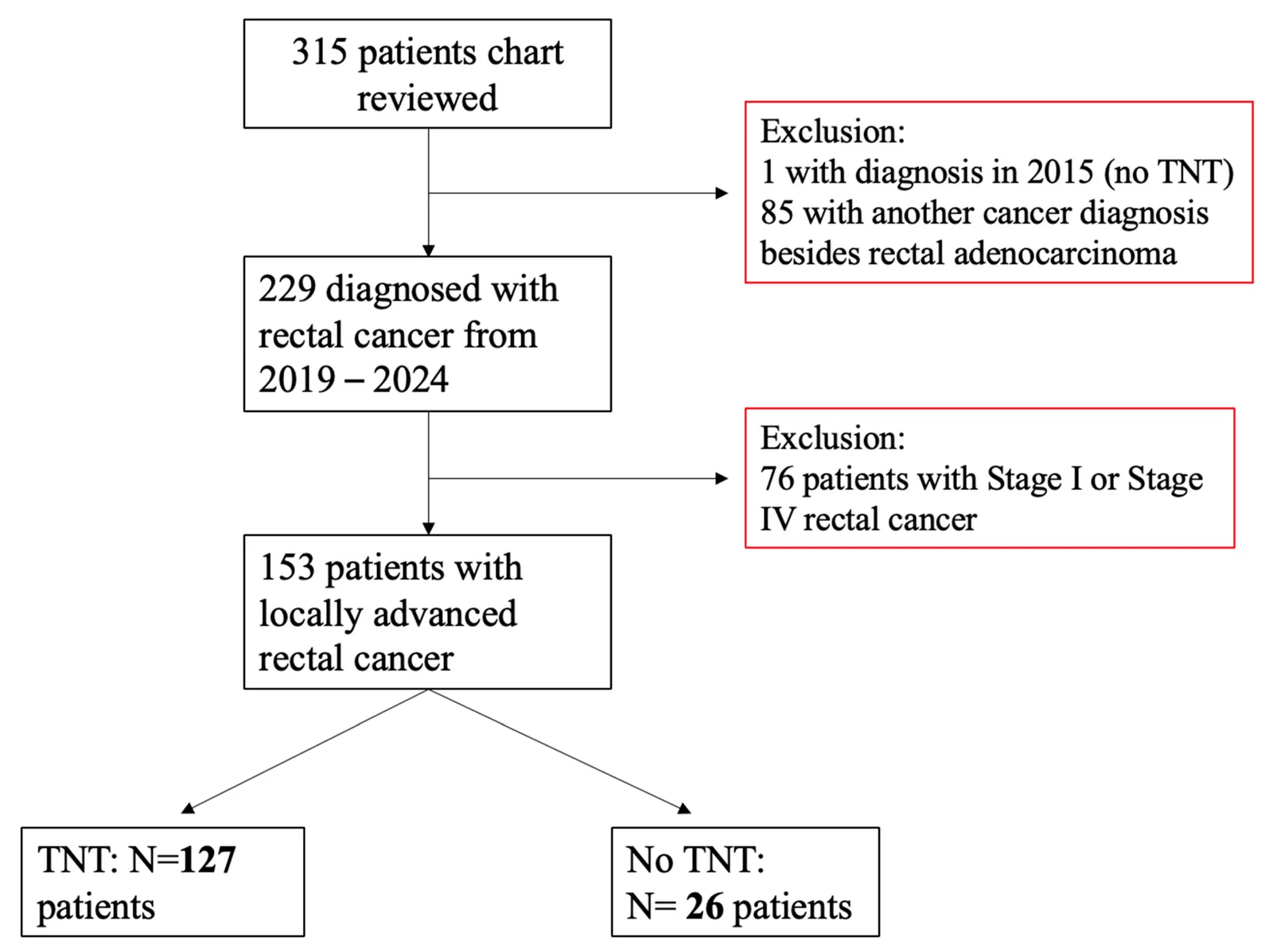
Flow diagram for patient inclusion. Abbreviations: TNT = Total Neoadjuvant Therapy.

Initially, the charts of 315 patients were reviewed, with the inclusion of 229 patients with rectal cancer diagnosed between the years 2019 and 2024. Eighty-five patients were excluded due to being presented at the tumor board but without a diagnosis of rectal adenocarcinoma. An additional patient was excluded as they were initially diagnosed with rectal cancer in 2015, prior to the implementation of TNT. One hundred fifty-three patients with locally advanced, non-metastatic rectal cancer (T3N0M0, TxN1-2M0) were included (Figure 2). A total of 127 patients underwent TNT treatment, and 26 patients did not undergo TNT treatment (Figure 2). Patient demographics for the patients who underwent TNT, and those who did not undergo TNT are listed in Table 2. There were statistically significant differences between the TNT and no TNT groups in the categories of ECOG status, location of the rectal tumor in regard to upper, middle, or low rectal cancers, and for treatment center type of academic vs. local centers. In Table 2, a delay in treatment initiation was chosen as a cut-off of greater than 8 weeks based on a study by Tevis et al., which demonstrated worse outcomes for patients who began adjuvant chemotherapy >8 weeks from the completion of surgery, with worse overall survival in addition to worse local and distant recurrence rates [10].

Of the patients who did not undergo TNT, reasons for not undergoing the typical treatment course included patient refusal of treatment, including chemotherapy and/or radiation, undergoing surgery first, or poor functional status of the patient.

Based on the TNT group, patients were more likely to undergo consolidation chemotherapy (74.8%) as opposed to induction chemotherapy (25.2%). The most used regimen for TNT was long-course chemoradiation followed by FOLFOX therapy. Given differences in treatment sites, initial biopsy features and patient baseline characteristics including baseline neuropathy and poor functional status, multiple other chemotherapy regimens were utilized including FOLFOXIRI (5-FU, leucovorin, irinotecan, oxaliplatin), FOLFIRI (5-FU, leucovorin, irinotecan), FOLFIRINOX (5-FU, leucovorin, irinotecan, oxaliplatin), 5-FU only, and 5-FU and leucovorin, in addition to capecitabine or CAPOX (Table 3). Given the variability in treatment at non-OHSU oncology centers, patients were typically prescribed anywhere from 6 to 8 cycles of systemic chemotherapy. After TNT completion, 70 patients underwent surgical intervention (low anterior resection or abdominoperineal resection), and 54 were enrolled in a watch-and-wait protocol. Forty-seven patients underwent surgery immediately after completing TNT, while 23 patients underwent surgery after a period of surveillance utilizing the watch-and-wait approach.

### 3.1. Adherence

Based on the 127 patients included who underwent TNT, fully captured data were available for 120 patients. One hundred four patients (86.6%) completed the entire course of TNT. There were 16 patients who did not complete the therapy. Within the induction chemotherapy group, there were 3 patients who did not tolerate their chemotherapy regimen prior to completing chemoradiation and another 3 who completed chemotherapy but could not complete the course of chemoradiation. Within the consolidation chemotherapy group, 9 patients completed their chemoradiation course but did not complete chemotherapy, and 1 patient did not complete chemoradiation but tolerated all their chemotherapy. Reasons for non-adherence included: admission for proctitis and bowel obstruction, intolerance to therapy, scheduling conflicts, anaphylactic reaction to the oxaliplatin, stroke, cytopenia, and patient request to stop therapy. Given the information available, the adherence rate to TNT therapy completion was 87.5%.

### 3.2. Adherence and Other Variables

#### 3.2.1. Age

Patients were categorized into age <50 years, 50–65 years, and >65 years. The adherence rates for each category were 90.6%, 83.3%, and 83.7%, respectively. There was no statistically significant difference between the different groups (*p* = 0.53) (Table 4).

#### 3.2.2. Gender

Female patients were reported to have an adherence rate of 78.7%, and male patients had an adherence rate of 91.8% (*p* = 0.04) (Table 4). The results are statistically significant within this category.

#### 3.2.3. Race

Most of the patients were White, which is in line with the institution’s state population. There is a small sample size for the remainder of the patients or missing data for reported race within the patients’ charts. The adherence rate for patients who were reported as White is 88.3%.

#### 3.2.4. Social History

Smoking and recreational drug use were assessed as they relate to adherence. Patients with a smoking history had an adherence rate of 83.8%, and non-smokers had an adherence rate of 90.2% (*p* = 0.42). Those with a history of recreational drug use had a lower adherence rate of 75.8% compared to those who did not have a history of drug use at 90.6% (*p* = 0.04) (Table 4). The results were statistically significant for favorable outcomes for non-recreational drug use history. There was no statistical significance with smoking history, but a trend that demonstrated improved adherence as a non-smoker.

#### 3.2.5. Facility Location and Type

Treatment facilities and patient proximity to the academic tertiary care center were assessed. The patients’ distance to the academic institution was grouped into those who lived within 50 miles of our institution and those who lived greater than 50 miles from our academic institution. The distance to Oregon Health and Science University (OHSU) did not appear to significantly affect adherence rates. Those living ≤50 miles from OHSU had an adherence rate of 88.4% and those >50 miles away had a rate of 85.7% (*p* = 0.78). The type of treatment facility did not appear to affect adherence, with adherence rates of 87.5% and 86.4% for academic and local community hospitals, respectively (*p* = 1.0).

#### 3.2.6. Eastern Cooperative Oncology Group (ECOG) Status

ECOG status measures functional status for cancer patients, with a score of 0 representing the ability to carry out all activities without restriction and a score of 3 representing a patient who only has limited ability to care for themselves and is confined to a bed or chair more than 50% of their waking hours. ECOG 0–1 was assessed together, and ECOG 2–3 were assessed together. Adherence rates for those with ECOG 0–1 were 90.5% and for ECOG 2–3 were 80.0% (*p* = 0.21). Although this was not statistically significant, it is clinically relevant as patients with worse performance status trended toward lower rates of adherence.

#### 3.2.7. Albumin

Albumin was assessed as ≤3 or >3, given the importance of albumin as a marker of nutritional status and as a determinant for primary anastomosis creation. Adherence rates for albumin ≤3 was 84.6%, while for patients with albumin >3 was 85.6% (*p* = 1.00). The albumin level is clinically relevant, especially for patients who require surgical intervention after completion of TNT.

#### 3.2.8. Sequence of Chemoradiation and Chemotherapy

**Figure 3:**
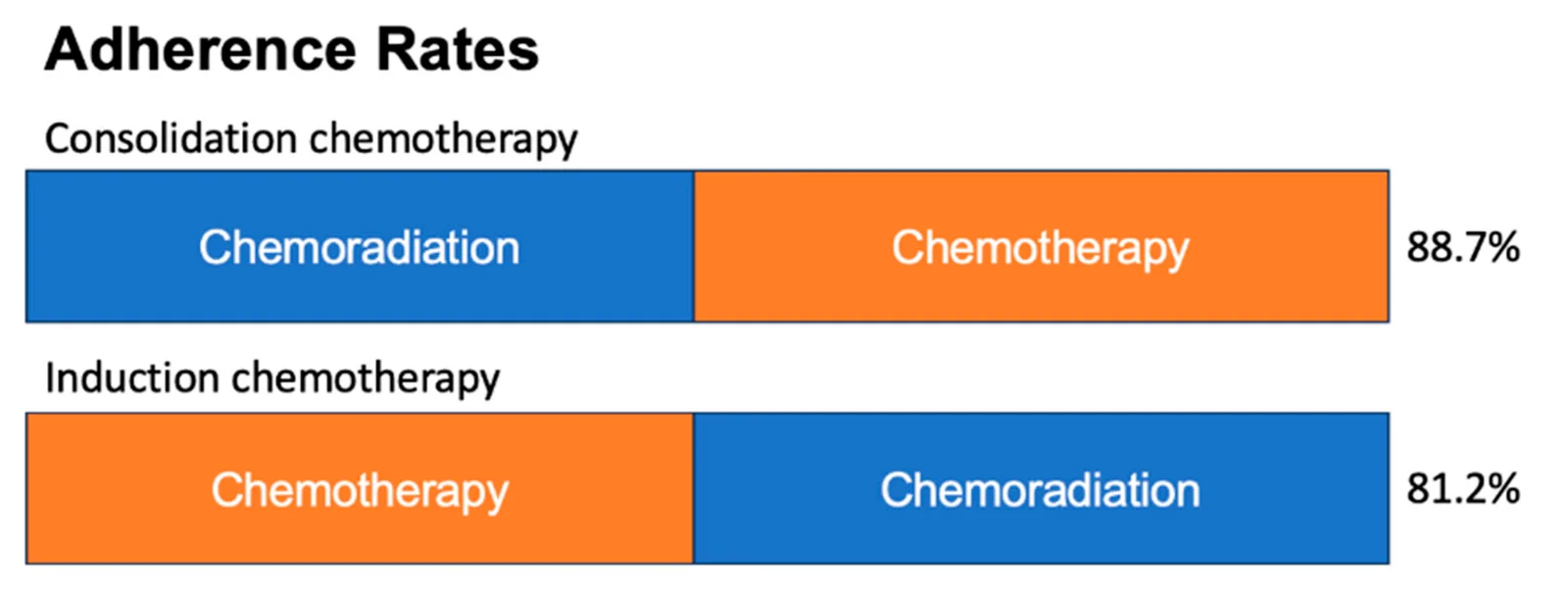
Adherence rates for consolidation chemotherapy (chemoradiation then chemotherapy) and induction chemotherapy (chemotherapy then chemoradiation).

The choice of induction versus consolidation chemotherapy did appear to impact adherence rates. For induction chemotherapy, the adherence rate was 81.2% and for consolidation chemotherapy, 88.7% (*p* = 0.36) (Figure 3). This was not statistically significant, but patients who received consolidation chemotherapy trended toward better adherence to therapy. Patients most often did not tolerate chemotherapy compared to chemoradiation (Table 5).

## 4. Discussion

This is one of the first real-world studies reporting adherence data for patients with LARC undergoing TNT therapy. A TNT adherence rate of 87.5% is reported in this study. Statistically significant differences between patients who underwent TNT and those who did not were observed for ECOG status, tumor location, and treatment center type. Patients who did not undergo TNT represented a smaller cohort and were more likely to have upper rectal tumors, which may have influenced the decision to proceed with a surgical intervention rather than TNT. Although most patients had an ECOG status of 0–1, those with poorer functional status with ECOG 2–3 may have influenced their treatment selection. Further investigation is needed to better delineate the association between treatment center type and TNT use.

We identified statistically significant differences in adherence for male patients and non-use of recreational drugs in patients who underwent TNT. We also observed trends among different demographics with improved adherence with consolidation chemotherapy and patients with improved performance status with ECOG scores of 0–1.

The observed 87.5% adherence rate has important implications for patient care. We understand that the historical adherence rates utilized a different treatment regimen and cannot be directly compared, but they do represent an improvement in treatment completion and potentially improved survival rates. The statistical significance of recreational drug non-use may be indicative of other patient factors, including socioeconomic status, mental health, social support, and difficulties with housing, which may also factor into treatment adherence. It is a variable that warrants further investigation, as it may be a surrogate for other challenges the patient may be facing.

A study by El Huseeini et al. [11] reported a retrospective chart review of patients treated at the American University of Beirut Medical Center with LARC. Their cohort included patients diagnosed from 2011 to 2019, with 81 patients diagnosed with LARC and 26 patients who received TNT. Their TNT therapy was not standardized and included both induction and consolidation chemotherapy with short-course radiotherapy followed by TME. The primary outcome of the study was to determine pathologic response, survival outcomes, and compliance with chemotherapy. They reported that all their patients in the TNT group began neoadjuvant chemotherapy and 84.6% completed the full course of treatment [11]. Their compliance rates for chemotherapy were similar to our findings. However, their small sample size of 26 patients did not allow for statistically meaningful analyses to evaluate variables for non-compliance, but cited reasons for non-compliance as postoperative complications, drug-related toxicities, and patient preferences.

The OPRA trial enrolled patients >18 years old with locally advanced rectal cancer and assessed organ preservation rates in patients with induction vs. consolidation chemotherapy. This study took place across 18 institutions with a trial protocol to ensure consistency. Their secondary analysis assessed compliance with TNT and looked at differences between induction and consolidation chemotherapy, although their study was not powered for these results. Their results demonstrated that patients were more likely to complete chemotherapy if they were randomized to the induction chemotherapy group, with 99% of patients completing it, a 97% compliance rate for the chemoradiation, and 93% of patients actually starting chemoradiation. In the consolidation chemotherapy group, 83% of patients completed FOLFOX, and 77% completed CAPEOX, with 98% completing chemoradiation [12]. Their compliance rates are better than expected, likely secondary to the risk of bias introduced by a randomized controlled trial compared to a retrospective real-world cohort. This can be due to a protocol set forth by the trial amongst all treating institutions, the instituted follow-up, and the observer effect of enrolling within a trial compared to a real-world retrospective study.

Our study has several limitations, including a small sample size from a single institution. Within the sample, a small percentage of this cohort was deemed non-adherent, which makes it harder to interpret the associations of the clinical variables and harder to detect a real difference. Given the limited patient population, multivariable regression was not performed as it would lead to overfitting of the model. The use of univariable analysis does not account for potential confounding among the variables. The retrospective study design also introduces missing data, including the inability to access patient records due to differences in the electronic medical records across institutions and undocumented or inconsistent reporting of some of the variables assessed. Selection bias is also possible as patients were identified through the multidisciplinary tumor board’s records. By utilizing the tumor board list, we may be excluding patients with rectal cancer who were seen at OHSU and not presented at our GI oncology tumor board, although per our institution’s National Accreditation Program for Rectal Cancer (NAPRC) guidelines, all patients with rectal cancer must be presented at our tumor board.

Oregon consists of a largely rural population, and most patients lived over 50 miles from our institution and were treated with TNT at local community hospitals. There was no single protocol instituted among treatment sites, resulting in variation in chemotherapy regimens and the number of treatment cycles received. The majority of the patients did utilize FOLFOX and consolidation chemotherapy, while the smaller number of patients receiving alternative regimens precluded meaningful comparison. Finally, the results of the study may not be generalizable outside of our institution to more racially and demographically diverse populations, given that a large proportion of patients in Oregon are white and live in a rural setting.

Future studies include increasing the current sample size with multicenter data sharing for a collaborative study. Another goal is designing prospective studies for patients undergoing TNT to determine adherence, along with assessing outcomes of pathological complete response, disease-free survival, overall survival, and disease recurrence with the inclusion of a large cohort of patients to perform multivariable analysis.

## 5. Conclusions

In conclusion, our data demonstrate that adherence rates with TNT are superior (87.5%) to the historically reported rates with the standard of care regimen including NACRT/TME/AC (40–57%). Statistically significant patient factors that support adherence in completing TNT include male gender and non-use of recreational drugs. These data may support how physicians evaluate behavioral patient characteristics to tailor their approach to counseling for cancer therapy.

## Figures and Tables

**Table 1:** Description of Rectal Cancer Treatment Trials: Trials that included adjuvant chemotherapy and observation (EORTC 22921 and CHRONICLE) and the trials instrumental in the implementation of total neoadjuvant therapy (TNT) (RAPIDO, PRODIGE-23). Abbreviations utilized: RT = radiation therapy, 5-FU = 5-fluorouracil, CAPOX = capecitabine and oxaliplatin, FOLFOX = 5-FU, oxaliplatin, leucovorin, FOLFIRINOX = 5-FU, oxaliplatin, leucovorin, irinotecan, NACRT = neoadjuvant chemoradiation, CRT = chemoradiation, TME = total mesorectal excision.

Trial	Experimental Treatment	Control	Key Result
**EORTC 22921**	Preop RT ± concurrent 5-FU → surgery → Adjuvant chemo	Preop RT ± concurrent 5-FU → surgery → Observation	No significant disease-free survival or overall survival benefit from adjuvant chemotherapy
**CHRONICLE**	NACRT → Surgery → Adjuvant CAPOX ×6	NACRT → Surgery → Observation	Compliance rate: 48% No significant disease-free survival benefit with adjuvant chemotherapy
**RAPIDO**	Short-course CRT → CAPOX ×6 or FOLFOX ×9 → TME	Long-course CRT → TME → Adjuvant chemo	Experimental group with an improved pathologic response rate and lower distant metastases
**UNI-CANCER-PRODIGE-23**	FOLFIRINOX ×6 → CRT → TME → Adjuvant chemo	CRT → TME → Adjuvant chemo	Experimental group with improved disease-free survival and pathological complete response rate

**Table 2:** Patient demographics for those who underwent TNT vs. those who did not undergo TNT. Averages are reported as mean (standard deviation). The bold characters in the first row signify the major categories of demographics assessed. The bold font in the last *p* value column represents a *p* value that is statistically significant, *p* < 0.05 between the two groups. Abbreviations: TNT = total neoadjuvant therapy, ECOG = Eastern Cooperative Oncology Group, OHSU = Oregon Health and Science University.

	TNT (*n* = 127)	No TNT (*n* = 26)	*p* Value *
**Age range, years**	35–92	37–85	
**Average age mean (SD), years**	61.4 (13.1)	63.1 (13.3)	0.57
**Gender**	Male 79 (62.2%)	Male 14 (53.8%)	0.57
**Race**			0.24
African American	0 (0.0%)	1 (3.9%)	
American Indian/Alaskan Native	3 (2.4%)	1 (3.9%)	
Asian	11 (8.6%)	2 (7.7%)	
Hispanic	3 (2.4%)	0 (0.0%)	
Non-Hispanic	3 (2.4%)	2 (7.7%)	
Pacific Islander	1 (0.8%)	0 (0.0%)	
White	105 (82.6%)	17 (65.3%)	
Unknown/Declined to answer	11 (8.6%)	3 (11.5%)	
**Smoking history**	Positive: 68 (53.5%)	Positive: 13 (50.0%)	0.91
**Stage**			0.20
IIA	31 (24.4%)	8 (30.8%)	
IIB	2 (1.6%)	2 (7.7%)	
IIC	9 (7.1%)	0 (0.0%)	
IIIA	10 (7.9%)	3 (11.5%)	
IIIB	52 (40.9%)	11 (42.3%)	
IIIC	23 (18.1%)	2 (7.7%)	
**Delay in initiation of treatment (>8 weeks)**	33 (26.0%)	10 (38.5%)	0.30
**ECOG**			**0.01**
0	55 (43.4%)	13 (50.0%)	
1	45 (35.4%)	4 (15.4%)	
2	15 (11.8%)	1 (3.9%)	
3	1 (0.8%)	3 (11.5%)	
Missing	11 (8.6%)	5 (19.2%)	
**Average CEA, median (IQR)**	2.9 (1.7–6.1)	2.3 (1.3–4.8)	0.42
**Rectum location**			**<0.001**
Upper	18 (14.2%)	14 (53.9%)	
Middle	60 (47.2%)	7 (26.9%)	
Low	49 (38.6%)	5 (19.2%)	
**Treatment Center**			**0.03**
Academic	33 (26.0%)	11 (42.3%)	
Local Community	94 (74.0%)	14 (53.9%)	
Missing	0 (0.0%)	1 (3.9%)	
**Distance from OHSU**			0.54
<25 miles	38 (29.9%)	8 (30.8%)	
26–50 miles	8 (6.3%)	3 (11.5%)	
>50 miles	81 (63.8%)	15 (57.7%)	

* Student *t*-test or Mann–Whitney U (rank-sum) test for continuous variables, Chi-square test or Fisher’s exact test for categorical variables when appropriate.

**Table 3:** TNT characteristics including type of TNT utilized, the systemic chemotherapy regimens utilized, radiation type, and information about surgical interventions.

TNT CHARACTERISTICS	
TNT TYPE	
INDUCTION	32 (25.2%)
CONSOLIDATION	95 (74.8%)
SYSTEMIC CHEMOTHERAPY REGIMEN	
FOLFOX	99 (77.9%)
CAPOX	14 (11.0%)
CAPECITABINE	6 (4.7%)
FOLFOXIRI	2 (1.6%)
FOLFIRINOX	2 (1.6%)
FOLFIRI	1 (0.8%)
5-FU LEUCOVORIN	1 (0.8%)
5-FU	2 (1.6%)
RADIATION TYPE	
LONG-COURSE	112 (88.2%)
SHORT-COURSE	15 (11.8%)
SURGICAL INTERVENTION	70 (55.1%)
TIMING OF SURGICAL INTERVENTION	
IMMEDIATELY AFTER TNT	47 (67.1%)
AFTER PERIOD OF WATCH AND WAIT	23 (32.9%)
AVERAGE TIME OF WATCH AND WAIT AFTER TNT COMPLETION (SD)	12.75 months (7.9)

**Table 4:** Characteristics and TNT therapy adherence. The bold font in the first column represents each of the characteristics assessed in the TNT group. In the *p*-value column the bold font represents a statistically significant finding with *p* < 0.05. Abbreviations: TNT = total neoadjuvant therapy, ECOG = Eastern Cooperative Oncology Group, OHSU = Oregon Health and Science University.

	Non-Adherence (*n* = 16)	Adherence (*n* = 104)	*p*-Value *
**N**	16	104	
**Age**			
<50	5	48	0.53
50–65	4	20	
>65	7	36	
**Race**			
White	12	91	1.00
Other	0	5	
**Gender**			
Female	10	37	**0.04**
Male	6	67	
**Smoking history**			
No	5	46	0.42
Yes	11	57	
**Recreational drug use history**			
No	8	77	**0.04**
Yes	8	25	
**Distance to OHSU**			
≤50 miles	5	38	0.78
>50 miles	11	66	
**Treatment center**			
Academic	4	28	1.00
Local	12	76	
**Albumin**			
≤3.0	2	11	1.00
>3.0	14	83	
**ECOG**			
0–1	9	86	0.21
2–3	3	12	
**TNT type**			
Induction	6	26	0.36
Consolidation	10	78	

* Student *t*-test or Mann–Whitney U (rank-sum) test for continuous variables, Chi-square test or Fisher’s exact test for categorical variables when appropriate. Note: Missing data are not included in the table.

**Table 5:** Adherence by TNT type (induction or consolidation).

Induction (N = 32)	Chemotherapy	Chemoradiation	*n* (%)
	×	×	0 (0%)
	√	×	3 (9.4%)
	×	√	3 (9.4%)
	√	√	26 (81.2%) *
Consolidation (N = 88)	Chemoradiation	Chemotherapy	*n* (%)
	×	×	0 (0%)
	√	×	9 (10.2%)
	×	√	1 (1.1%)
	√	√	78 (88.7%) *

* Note: Adherence to both chemoradiation and chemotherapy indicates full adherence with TNT therapy. Legend: √ = Adherence, × = Non-adherence. Colors of blue and orange chosen to demonstrate consistency with the next figure, there is no association of the colors with the treatment regimen.

## Data Availability

The data presented in this study are available on request from the corresponding author because it contains patient-identifying information.

## Abbreviations

LARC	Locally advanced rectal cancer
NACRT	Neoadjuvant chemoradiation
TME	Total mesorectal excision
AC	Adjuvant chemotherapy
TNT	Total neoadjuvant therapy
FOLFOX	5-Fluorouracil, Leucovorin, Oxaliplatin
5-FU	5-Fluorouracil
FOLFIRINOX	5-Fluorouracil, oxaliplatin, leucovorin, irinotecan
FOLFOXIRI	5-Fluorouracil, leucovorin, irinotecan, oxaliplatin
FOLFIRI	5-Fluorouracil, leucovorin, irinotecan
CAPOX	Capecitabine, Oxaliplatin
REDCap	Research Electronic Data Capture
ECOG	Eastern Cooperative Oncology Group
OHSU	Oregon Health and Science University

## References

[1] Bosset J.F., Collette L., Calais G., Mineur L., Maingon P., Radosevic-Jelic L., Daban A., Bardet E., Beny A., Ollier J.C. (2006). Chemotherapy with preoperative radiotherapy in rectal cancer. N. Engl. J. Med..

[2] Glynne-Jones R., Counsell N., Quirke P., Mortensen N., Maraveyas A., Meadows H.M., Ledermann J., Sebag-Montefiore D. (2014). Chronicle: Results of a randomised phase III trial in locally advanced rectal cancer after neoadjuvant chemoradiation randomising postoperative adjuvant capecitabine plus oxaliplatin (XELOX) versus control. Ann. Oncol..

[3] Bosset J.F., Calais G., Mineur L., Maingon P., Stojanovic-Rundic S., Bensadoun R.J., Bardet E., Beny A., Ollier J.C., Bolla M. (2014). Fluorouracil-based adjuvant chemotherapy after preoperative chemoradiotherapy in rectal cancer: Long-term results of the EORTC 22921 randomised study. Lancet Oncol..

[4] Mari G.M., Maggioni D., Crippa J., Costanzi A.T.M., Scotti M.A., Giardini V., Garancini M., Cocozza E., Borroni G., Benzoni I. (2019). Compliance to Adjuvant Chemotherapy of Patients Who Underwent Surgery for Rectal Cancer: Report from a Multi-institutional Research Network. World J. Surg..

[5] Garcia-Aguilar J., Chow O.S., Smith D.D., Marcet J.E., Cataldo P.A., Varma M.G., Kumar A.S., Oommen S., Coutsoftides T., Hunt S.R. (2015). Effect of adding mFOLFOX6 after neoadjuvant chemoradiation in locally advanced rectal cancer: A multicentre, phase 2 trial. Lancet Oncol..

[6] Bahadoer R.R., Dijkstra E.A., van Etten B., Marijnen C.A., Putter H., Meershoek-Klein Kranenbarg E., Roodvoets A.G.H., Nagtegaal I.D., Beets-Tan R.G.H., Blomqvist L.K. (2021). Short-course radiotherapy followed by chemotherapy before total mesorectal excision (TME) versus preoperative chemoradiotherapy, TME, and optional adjuvant chemotherapy in locally advanced rectal cancer (RAPIDO): A randomised, open-label, phase 3 trial. Lancet Oncol..

[7] Conroy T., Bosset J.F., Etienne P.L., Rio E., Francois E., Mesgouez-Nebout N., Vendrely V., Artignan X., Bouche O., Gargot D. (2021). Neoadjuvant chemotherapy with FOLFIRINOX and preoperative chemoradiotherapy for patients with locally advanced rectal cancer (UNICANCER-PRODIGE 23): A multicentre, randomised, open-label, phase 3 trial. Lancet Oncol..

[8] Garcia-Aguilar J., Patil S., Gollub M.J., Kim J.K., Yuval J.B., Thompson H.M., Verheij F.S., Omer D.M., Lee M., Dunne R.F. (2022). Organ Preservation in Patients With Rectal Adenocarcinoma Treated With Total Neoadjuvant Therapy. J. Clin. Oncol..

[9] Harris P.A., Taylor R., Thielke R., Payne J., Gonzalez N., Conde J.G. (2009). Research electronic data capture (REDCap)–A metadata-driven methodology and workflow process for providing translational research informatics support. J. Biomed. Inform..

[10] Tevis S.E., Kohlnhofer B.M., Stringfield S., Foley E.F., Harms B.A., Heise C.P., Kennedy G.D. (2013). Postoperative complications in patients with rectal cancer are associated with delays in chemotherapy that lead to worse disease-free and overall survival. Dis. Colon. Rectum..

[11] Husseini Z.E., Haibe Y., Bouferraa Y., Kreidieh M., Al Darazi M., Mukherji D., Temraz S., Charafeddine M., Shamseddine A. (2021). Total neoadjuvant therapy in patients with locally advanced rectal cancer: A tertiary medical center experience. Mol. Clin. Oncol..

[12] Verheij F.S., Omer D.M., Lin S.T., Yuval J.B., Thompson H.M., Kim J.K., Valdivieso S.C., Qin L.X., Wu A.J., Saltz L.B. (2024). Compliance and Toxicity of Total Neoadjuvant Therapy for Rectal Cancer: A Secondary Analysis of the OPRA Trial. Int. J. Radiat. Oncol. Biol. Phys..

